# Oral *Clostridium butyricum* on mice endometritis through uterine microbiome and metabolic alternations

**DOI:** 10.3389/fmicb.2024.1351899

**Published:** 2024-02-21

**Authors:** Mao Hagihara, Tadashi Ariyoshi, Shuhei Eguchi, Kentaro Oka, Motomichi Takahashi, Hideo Kato, Yuichi Shibata, Takumi Umemura, Takeshi Mori, Narimi Miyazaki, Jun Hirai, Nobuhiro Asai, Nobuaki Mori, Hiroshige Mikamo

**Affiliations:** ^1^Department of Molecular Epidemiology and Biomedical Sciences, Aichi Medical University, Nagakute, Japan; ^2^Department of Clinical Infectious Diseases, Aichi Medical University, Nagakute, Japan; ^3^R&D Division, Miyarisan Pharmaceutical Co., Ltd., Saitama, Japan

**Keywords:** *Clostridium butyricum*, endometritis, microbiome, metabolome, resolvin D5, *Lactobacillus* species, *Limosilactobacillus* species, G protein-coupled receptor 120

## Abstract

Endometritis occurs frequently in humans and animals, which can negatively affect fertility and cause preterm parturition syndrome. Orally administered *Clostridium butyricum*, a butyrate-producing gram-positive anaerobe, exhibits anti-inflammatory effects. However, the precise mechanism by which *Clostridium butyricum* attenuates endometritis remains unclear. This *in vivo* study evaluated the anti-inflammatory effects of orally administered *Clostridium butyricum* on uterine tissues. In addition, we conducted uterine microbiome and lipid metabolome analyses to determine the underlying mechanisms. Female Balb/c mice were divided into the following four groups (*n* = 5–20): (1) mock group, (2) only operation group (mice only underwent operation to exposed uterine horns from the side), (3) control group (mice underwent the same operation with the operation group + perfusion of lipopolysaccharide solution from uterine horns), and (4) *Clostridium butyricum* administration group (mice underwent the same operation with the control group + oral *Clostridium butyricum* administration from days 0 to 9). *Clostridium butyricum* was administered via oral gavage. On day 10, we investigated protein expression, uterine microbiome, and lipid metabolism in uterine tissues. Consequently, orally administered *Clostridium butyricum* altered the uterine microbiome and induced proliferation of *Lactobacillus* and *Limosilactobacillus* species. The effects can contribute to show the anti-inflammatory effect through the interferon-β upregulation in uterine tissues. Additionally, oral *Clostridium butyricum* administration resulted in the upregulations of some lipid metabolites, such as ω-3 polyunsaturated fatty acid resolvin D5, in uterine tissues, and resolvin D5 showed anti-inflammatory effects. However, the orally administered *Clostridium butyricum* induced anti-inflammatory effect was attenuated with the deletion of G protein-coupled receptor 120 and 15-lipooxgenase inhibition. In conclusion, *Clostridium butyricum* in the gut has anti-inflammatory effects on uterine tissues through alterations in the uterine microbiome and lipid metabolism. This study revealed a gut-uterus axis mechanism and provided insights into the treatment and prophylaxis of endometritis.

## Highlights

- Orally administered *Clostridium butyricum* induces the proliferation of *Lactobacillus* and *Limosilactobacillus* species in the uterine microbiome.
- Oral *Clostridium butyricum* administration results in the alternations of lipid metabolisms in uterine tissues.
- Orally administered *Clostridium butyricum* shows anti-inflammatory effects through G protein-coupled receptor 120 and the interferon-β upregulation in uterine tissues.

## 1 Introduction

Endometritis occurs frequently in humans and animals, which can negatively affect fertility (Ravel et al., 2021). Additionally, among the pathological processes implicated in preterm parturition syndrome, endometritis is one of the main causes (Romero et al., 2006; Cicinelli et al., 2008; Goldenberg et al., 2008; Saito et al., 2010), and the disease is mainly caused by the invasion of pathogenic microorganisms such as *Escherichia coli* and *Bacteroides* species (Wagener et al., 2014; Wang et al., 2018; Galvão et al., 2019; Pascottini et al., 2020; Paiano et al., 2021).

The uterus is not sterile, and a microbiome exists inside the uterus (Lozano et al., 2021). The commensal microbiome in the uterus contributes to many host physiological processes that maintain homeostasis by affecting the immune system and metabolic activities (Hooper et al., 2012). However, endometritis causes an imbalance in bacterial composition (dysbiosis) in the uterine microbiome (Moreno et al., 2016; Lozano et al., 2021), which triggers a host pro-inflammatory immune response and induces various inflammatory and metabolic diseases (Garrett et al., 2007; Vijay-Kumar et al., 2010; Elinav et al., 2011). Notably, dysbiosis of not only the vaginal microbiome but also the uterine microbiome affects pregnancy and birth rates (Moreno et al., 2016; Liu et al., 2019).

Systemic antibiotics are used to treat endometritis; however, their efficacy on the birth rate remains controversial (Cicinelli et al., 2015; Yoneda et al., 2016; Kuroda et al., 2021; Kato et al., 2022), and only limited therapies for the prevention and treatment of endometritis are available. Furthermore, similar to other organs, systemic antibiotics can affect physiological processes by altering the uterine microbiome alternations (Hagihara et al., 2020; Gao et al., 2023). Therefore, identifying alternative and safe ways to treat endometritis is important, since even partially effective therapies can increase birth rates.

*Clostridium butyricum* (ATCC 19398), a butyrate-producing gram-positive anaerobe, shows anti-inflammatory effects against endometritis when administered directly into the uterus (Mun et al., 2022). However, current probiotic dosing directly into the uterus is not realistic owing to technical issues in clinical situations, and the effects of orally administered *C. butyricum* on endometritis remain unclear.

*Clostridium butyricum* MIYAIRI 588 (CBM 588) has been used to treat gastrointestinal symptoms, such as diarrhea, as probiotics in Japan (Seki et al., 2003). Orally administered CBM 588 protects against intestinal necrosis and inflammation caused by antibiotic administration and *Clostridioides difficile* infection, by altering the gut microbiome and metabolic functions (Hagihara et al., 2018, 2020, 2021; Ariyoshi et al., 2020, 2021). Additionally, orally administered CBM 588 attenuates lung inflammation by upregulating lipid metabolites produced in the gut (Hagihara et al., 2022). Hence, oral CBM 588 administrations can show anti-inflammatory effects not only in the gut, but also in other tissues separate from the gut, such as the uterus.

Therefore, this *in vivo* study aimed to evaluate whether orally administered CBM 588 has anti-inflammatory effects in uterine tissues. In addition, we conducted uterine microbiome and lipid metabolome analyses to determine the underlying mechanisms.

## 2 Materials and methods

### 2.1 Mice

Pathogen-free female Balb/c and C57BL/6 J mice (8–10 weeks) weighing approximately 20–23 g were used (Charles River Laboratories Japan, Inc., Yokohama, Japan). *Gpr120* −/− mice (RBRC10195) and *Irf-7* −/− mice (RBRC01420) were purchased from RIKEN BioResource Research Center. As previously described, the mice were provided with food and water *ad libitum* (Hagihara et al., 2022). This *in vivo* study was performed in accordance with the ARRIVE guidelines 2.0,^1^ American Veterinary Medical Association guidelines,^2^ and the Japanese College of Laboratory Animal Medicine’s publication guide.^3^ The Ethics Committee of Aichi Medical University reviewed and approved this study (2023-58).

### 2.2 Treatments

Female Balb/c mice were divided into the following four groups: (1) mock group, (2) only operation group (mice only underwent operation to exposed uterine horns from the side), (3) control group (mice underwent the same operation with the operation group + perfusion of lipopolysaccharide [LPS] solution from uterine horns), and 4) CBM 588 administration group (mice underwent same operation with the operation group + perfusion of LPS solution from uterine horns + oral CBM 588 administration from day 0 to 9. Under anesthesia, mice were injected with 100 μL LPS (Sigma, L2880) solution (2.5 mg/mL in phosphate-buffered saline [PBS]) into each uterine horn through a micro syringe on days 0 and 5 (Jiang et al., 2021; Shaukat et al., 2021). CBM 588 (2.2 × 10^10^ CFU/g: Lot 61GT) was administered by oral gavage at 500 mg/kg/day (3.4 × 10^8^ CFU/kg/day). Resolvin D5 (Cayman, 100,072,809) at 0.3 μg/100 μL in PBS and PD146176 (S6956^4^) at 0.3 mg/300 μL in PBS were administered to mice intraabdominally (Gobbetti et al., 2017; Ariyoshi et al., 2020).

### 2.3 Physiological condition assessment

During the study period, weight loss was monitored daily and reported as the percentage of weight loss from the initial body weight (day 0) (Hagihara et al., 2022). On day 10, the mice were euthanized with an overdose of CO_2_ followed by cervical dislocation. The uterus and colon were weighed. Additionally, uterine tissues were homogenized in RIPA buffer supplemented with protease inhibitors (Nacalai tesque, 08714-04). The suspension was centrifuged at 10,000 × *g* for 5 min, 4°C. Frozen supernatants from the mouse uterine tissue samples (stored −80°C) were thawed once to room temperature, and detected protein concentrations with ELISA kits (interferon (IFN)-β (MIFNB0) from RSD, tumor necrosis factor alpha (TNF-α) (430907), interleukin (IL)-10 (43147), IL-6 (431307), transforming growth factor (TGF)-β (436707), IFN-γ (430807), IL-17A (432507), and IL-4 (431417) from LegendMax) according to manufacturer’s guidance.

### 2.4 Pathologic evaluation

Harvested uterine tissues that were fixed with 10% neutral-buffered formalin were embedded in paraffin and cut into 3-μm sections, and stained with hematoxylin and eosin (H&E) for histological analysis via light microscopy (Hagihara et al., 2020). Uterine tissues were evaluated by a skilled pathologist.

### 2.5 Uterine microbiome analysis

Uterine microbiome analysis was conducted as described in our previous study (Hagihara et al., 2022), with minor modifications. Briefly, 100 μL PBS solution was perfused from one of two uterine horns with a microsyringe after tying the upper vagina tightly with a thread and the edge of the other side of uterine horn was cut. The PBS lavage fluid was used to characterize the microbiome composition of uterine tissues by sequencing the V3-V4 regions of the 16S rRNA gene. To extract DNA, the lavage fluid was suspended in 10 mM Tris–HCl and 10 mM EDTA buffer (pH 8.0), and added lysozyme (15 mg/mL; Sigma). After incubation at 37°C for 1 h, achromopeptidase (Wako) was added (2,000 U/mL) to the sample and incubated at 37°C for 30 min. As next step, SDS was added (1%) and mixed well. Then, proteinase K (Merck) was added (1 mg/mL) to the suspension and incubated at 55°C for 1 h. High-molecular mass DNA was isolated and purified by phenol/chloroform extraction, ethanol precipitation, and finally polyethylene glycol precipitation. For microbiome analysis, meta 16S rRNA gene sequencing PCR was performed by using Ex Taq Hot Start (TAKARA) and the Illumina forward primer 50-AATGATACGGCGACCACCGAGATCTACAC (adaptor sequence) + barcode (eight bases) + ACACTCTTTCCC TACACGACGCTCTTCCGATCT (sequence primer) + CCTACGGGNG GCWGCAG-30 (341F) and the Illumina reverse primer 50-CAAGCAGAAGACGGCATACGAGAT (adaptor sequence) + barcode (eight bases) + GTGACTGGAGTTCAGACGTGTGCTCTTCCGATCT (sequence primer) + GACTACHVGGGTATCTAATCC-30 (805R) to amplify the hypervariable V3-V4 region of the 16S rRNA gene. Amplicons generated from each sample were subsequently purified using SPRI select (Beckman Coulter). The amount of DNA was quantified using the QuantiFluor dsDNA System and a Quantus Fluorometer (Promega). Mixed samples were prepared by pooling approximately equal amounts of each amplified DNA and sequenced using the MiSeq Reagent Kit V3 (600 cycle) and a MiSeq sequencer (Illumina), according to the manufacturer’s instructions. The 16S rRNA sequence data generated by the MiSeq sequencer (Illumina) were processed using the quantitative insights into the microbial ecology (QIIME 2) pipeline as we previously published (Hagihara et al., 2022). Alpha- and β-diversity analysis were calculated using QIIME. An α-rarefaction curve was generated using the Chao 1 estimator of species richness. To compare the microbial composition between samples, β-diversity was measured by calculating the weighted UniFrac distances using QIIME default scripts. Principal coordinate analysis (PCoA) was applied to the resulting distance matrices of uterine microbiome at species level to generate two-dimensional plots.

### 2.6 Impact of *Clostridium butyricum* on Lactobacilluceae proliferation

*Lactobacillus* spp. (*L. jensenii* Ich 2023-1, *L. iners* 22-2590, *L. gasseri* Ich 2023-1, *L. crispatus* 8-1, *L. crispatus* 14-2), *Limosilactobacillus* spp. (*L. vaginalis* Ich9595, *L. reuteri* YB1506, *L. fermentum* YB1839) strains were cultured anaerobically (6% H_2_, 20% CO_2_, 74% N_2_ at 37°C) with shaking in GAM broth (Nissui Pharmaceutical Co., Ltd. 05422) containing supernatants of CBM 588 culture solution of GAM broth (Nissui Pharmaceutical Co., Ltd.) at 0, 1, 5, and 10%. Colony measurements were performed after 24 h incubations (*n* = 4 per group). To assess bacterial concentrations, samples were obtained and serially diluted in normal saline. Aliquots of the diluted samples were plated for quantitative culture. Brucella HK nutrient agar plates (100-mm diameter) were used for quantitative determinations (Kyokuto Pharmaceutical Industrial Co., Ltd). The colony counts were read after 48 to 72 h of incubation anaerobically at 37°C.

### 2.7 Long-chain lipid metabolome analysis

Long-chain lipid metabolic analyses were conducted (Ariyoshi et al., 2020, 2021; Hagihara et al., 2022). Briefly, uterine tissues were sampled from the mice and immediately cryopreserved (stored at −80°C). The uterine tissues were lyophilized and 10 mg was weighed after ball milling. Methanol (180 μL) was added, and the mixture was vortexed for 1 min. We then used a Vanquish UHPLC system (Thermo Fisher Scientific), Q Exactive Focus (Thermo Fisher Scientific) with an electrospray ionization device, and liquid chromatography–tandem mass spectrometry (LC–MS/MS) and Orbitrap LC–MS/MS analyses using an Acclaim RSLC120 C18 (Thermo Fisher Scientific) to conduct analysis.

### 2.8 Quantifications and statical analysis

Data are presented as mean ± standard deviation. Statistical analyses were performed using the GraphPad Prism 9 software (GraphPad Software, San Diego, CA, USA). Statistical significance was set at *p* < 0.05 (****p < 0.0001, ***p < 0.001, **p < 0.01, *p < 0.05; ns indicates not significant).

## 3 Results

### 3.1 Anti-inflammatory effects of orally administered *Clostridium butyricum* in uterine tissue

**Figure 1:**
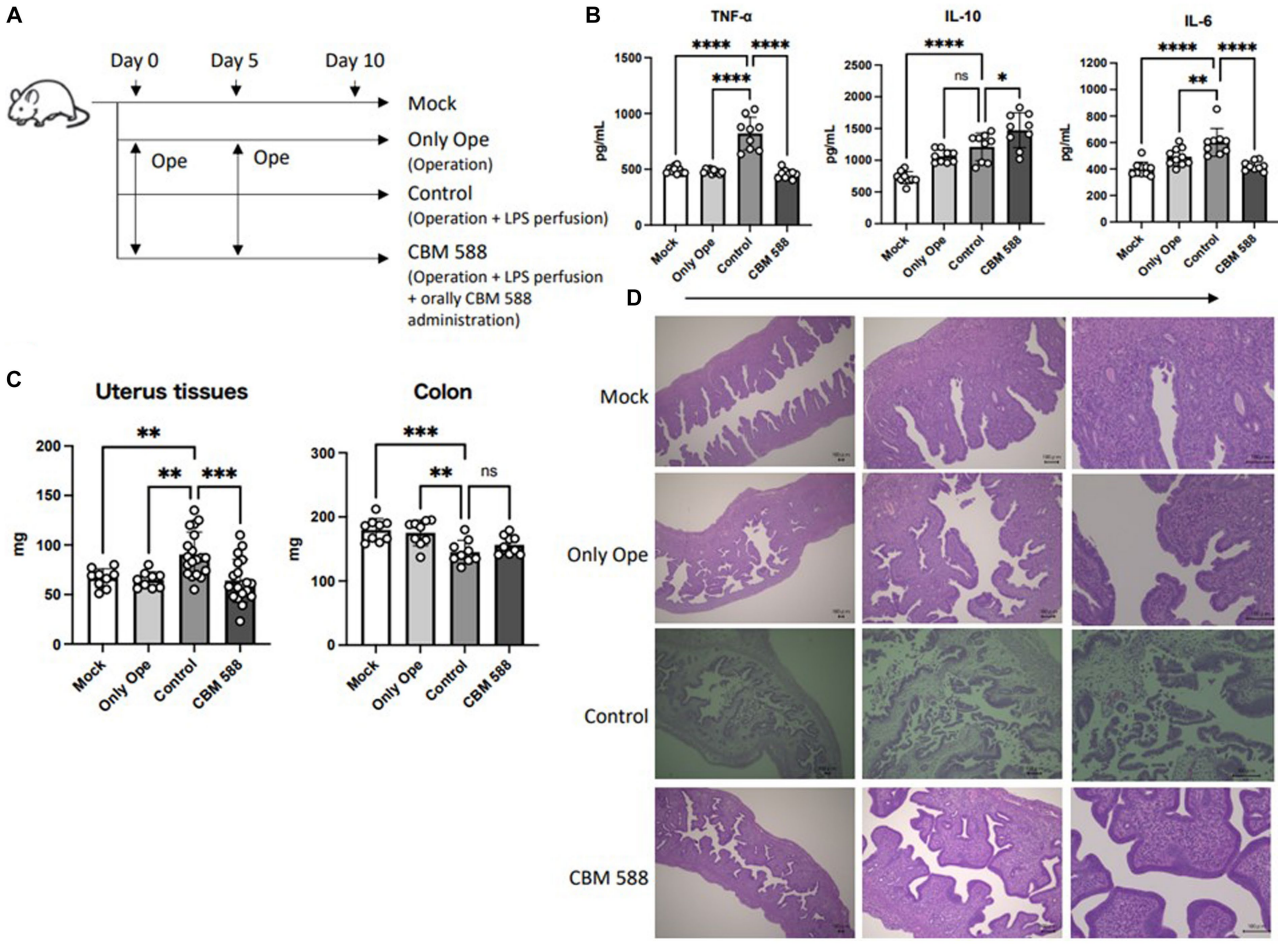
Anti-inflammatory effects of orally administered *Clostridium butyricum* in uterine tissue. **(A)** Balb/c mice were divided into 4 groups. Mock group received no treatments; only operation group, control group and CBM 588 group received operations on days 0 and 5. Control group and CBM 588 group conducted LPS perfusions from the uterus horns. Only CBM 588 groups received oral CBM 588 administrations from days 0 to 9. **(B)** Cytokine levels in uterine tissues on day 10 (*n* = 9 or 10). **(C)** Weights of uterine tissues (*n* = 9 or 19) and colon tissues (*n* = 9). **(D)** Representative histological images of uterine tissues on day 10 (scale bar, 100 mm, bottom right). Results are presented as mean ± standard deviation. Each dot represents a single mouse. The results were considered statistically significant when the differences were *p* < 0.05, as determined using one-way ANOVA **(B,C)**. See also Supplementary Figure S1. CBM 588, *Clostridium butyricum* MIYAIRI 588; LPS, lipopolysaccharide.

To determine whether CBM 588 has anti-inflammatory effects, we orally administered CBM 588 to mice with LPS-induced endometritis (Figure 1A). On day 10, we observed an upregulation of pro-inflammatory cytokines in the control group compared to the mock and operation groups (Figure 1B). Conversely, the CBM 588 administration group showed lower pro-inflammatory cytokines, such as TNF-α, IL-17A, IFN-γ, and IL-6, and higher anti-inflammatory cytokines, such as IL-10, TGF-β and IL-4, in the uterine tissues than those of the control group (Figure 1B; Supplementary Figure S1A). Moreover, among mice with endometritis, the weight gain of uterine tissues was significantly attenuated by CBM 588 administration (Figure 1C), whereas the body weights and survival rates did not show significant differences between the control and CBM 588 groups (Supplementary Figures S1B,C). These findings and historical evaluations suggest that CBM 588 can elicit anti-inflammatory effects in uterine tissues by attenuating the inflammatory symptoms caused by endometritis (Figure 1D).

### 3.2 Effect of oral *Clostridium butyricum* on uterine microbiome

**Figure 2:**
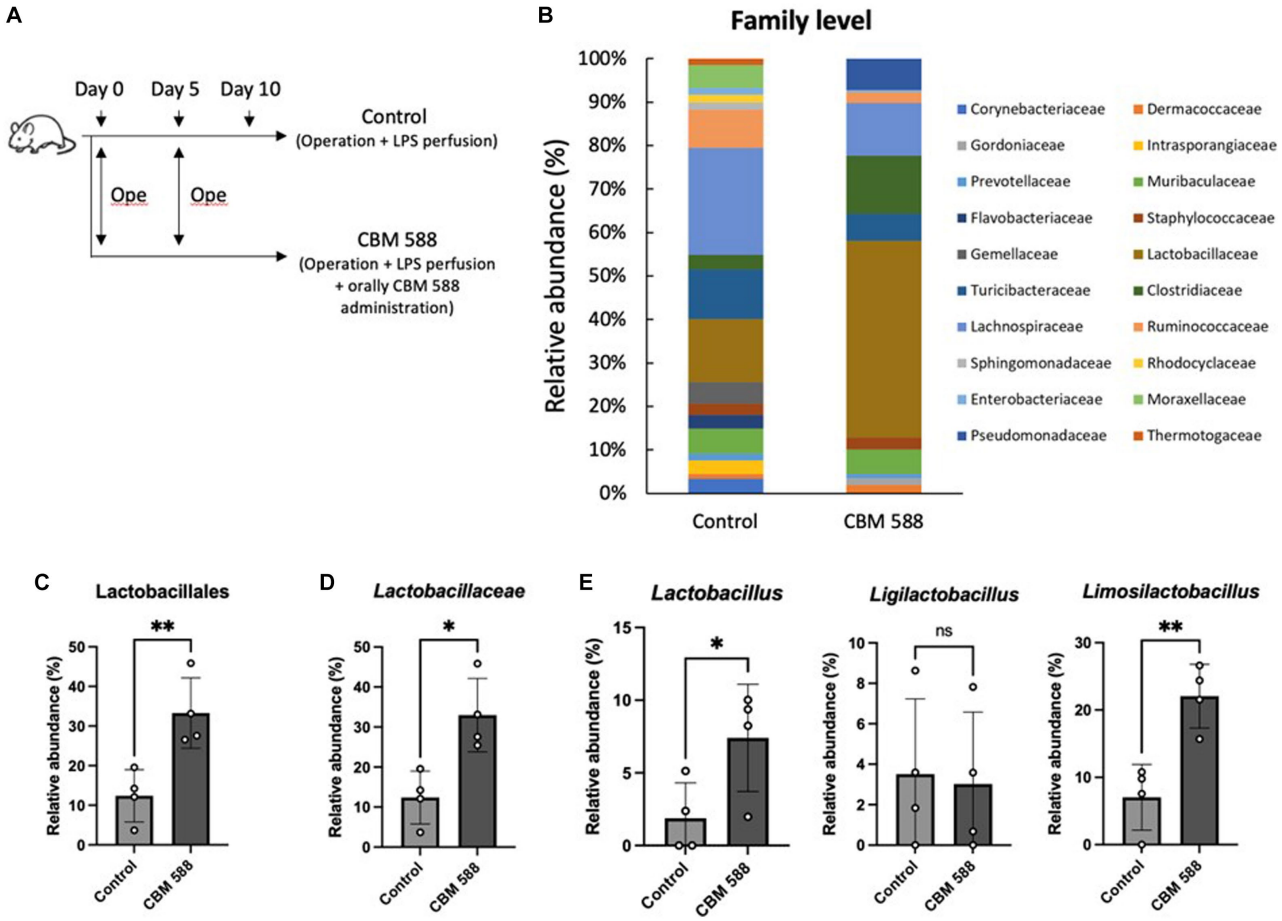
Effect of oral *C. butyricum* on uterine microbiome. **(A)** Balb/c mice were divided into 2 groups. Control group (*n* = 4) and CBM 588 group (*n* = 4) received operations on days 0 and 5. Control group and CBM 588 group conducted LPS perfusions from the uterus horns. Only CBM 588 group received oral CBM 588 administrations from days 0 to 9. **(B)** Bacterial composition in uterine tissues at the family level. **(C)** Relative abundances of Lactobacillales in uterine microbiome at order level. **(D)** Relative abundances of *Lactobacillaceae* in uterine microbiome at family level. **(E)** Relative abundances of *Lactobacillus*, *Ligilactobacillus*, and *Limosilactobacillus* in uterus microbiome at genera level. Results are presented as mean ± standard deviation. Each dot represents a single mouse. Results were considered statistically significant when the differences were *p* < 0.05, as determined by Student’s *t*-test **(C–E)**. See also Supplementary Figure S2. CBM 588, *Clostridium butyricum* MIYAIRI 588; LPS, lipopolysaccharide.

To reveal the effect of CBM 588 on uterine microbiomes, we performed uterine microbiome analysis among the control and CBM 588 administration group on day 10 (Figure 2A). The bar graphs depict the mean relative abundance of bacterial groups at the phylum to family levels (Figure 2B; Supplementary Figure S2A). CBM 588 administration did not significantly alter the relative abundances of each bacterial group at phylum and class levels compared to the control group (Supplementary Figure S2A). Additionally, CBM 588 administration did not significantly change α-diversity and β-diversity in the uterine microbiome compared with the control group (Supplementary Figures S2B,C). However, at the order and family levels, respectively, the CBM 588 administration group had significantly higher relative abundances of only Lactobacillales and *Lactobacillaceae*, compared with the control group (Figures 2C,D). Additionally, at the genus level, *Lactobacillu*s, *Ligilactobacillus*, and *Limosilactobacillus* were detected in the *Lactobacillaceae* group, and the CBM 588 administration group had significantly higher relative abundance of *Lactobacillus* and *Limosilactobacillus* than the control group (Figure 2E), whereas the other groups did not show the significantly differences.

### 3.3 Supernatant of *Clostridium butyricum* promotes *Lactobacillus* spp. and *Limosilactobacillus* spp. proliferations

**Figure 3:**
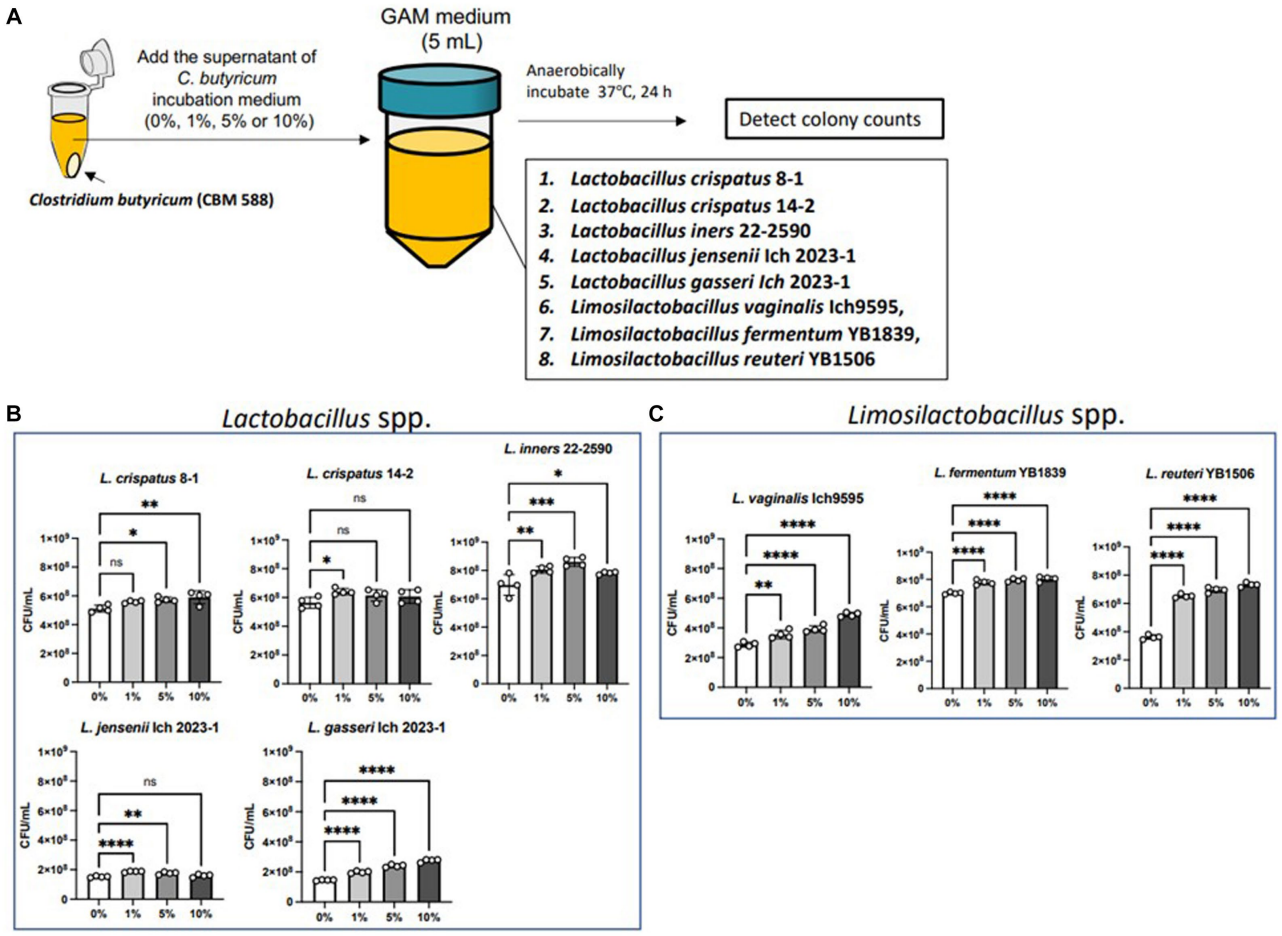
Supernatant of *C. butyricum* promotes *Lactobacillus* spp. and *Limosilactobacillus* spp. proliferations. **(A)** *Lactobacillus* spp. and *Limosilactobacillus* spp. were exposed to the supernatant of *C. butyricum* incubation medium (0, 1, 5, and 10%) and incubated anaerobically for 24 h. **(B)** Bacterial concentrations of *Lactobacillus* spp. (*n* = 4, respectively). **(C)** Bacterial concentrations of *Limosilactobacillus* spp. (*n* = 4, respectively). Results are presented as mean ± standard deviation. Each dot represents a single sample. The results were considered statistically significant when the differences were *p* < 0.05, as determined using one-way ANOVA **(B,C)**. See also Supplementary Figure S3. CBM 588, *Clostridium butyricum MIYAIRI* 588.

We conducted an *in vitro* study to determine the effects of CBM 588 on the proliferation of *Lactobacillus* and *Limosilactobacillus* spp. (Figure 3A). We evaluated seven species (eight strains) that were frequently detected in the organs of the genus organs (Haakensen et al., 2011; Duar et al., 2017; Liu and Gu, 2020; Zheng et al., 2020; Wang J. et al., 2023). CBM 588 supernatant enhanced *L. gasseri, L. vaginalis, L. fermentum*, and *L. reuteri* proliferations in a dose-dependent manner (Figures 3B,C). They showed significantly enhanced proliferations in the GAM broth after 24 h incubation anaerobically, even though the bacterial suspensions were containing only 1% of the CBM 588 culture medium supernatant. Among the eight isolates, *L. reuteri* showed the highest ratio of the bacterial concentrations (bacteria suspensions containing CBM 588 supernatants at 1, 5, and 10% /bacterial suspensions without CBM 588 supernatants [0%]) (Supplementary Figure S3A).

### 3.4 Orally administered *Clostridium butyricum* shows anti-inflammatory effects through the interferon-β upregulation in uterine tissues

**Figure 4:**
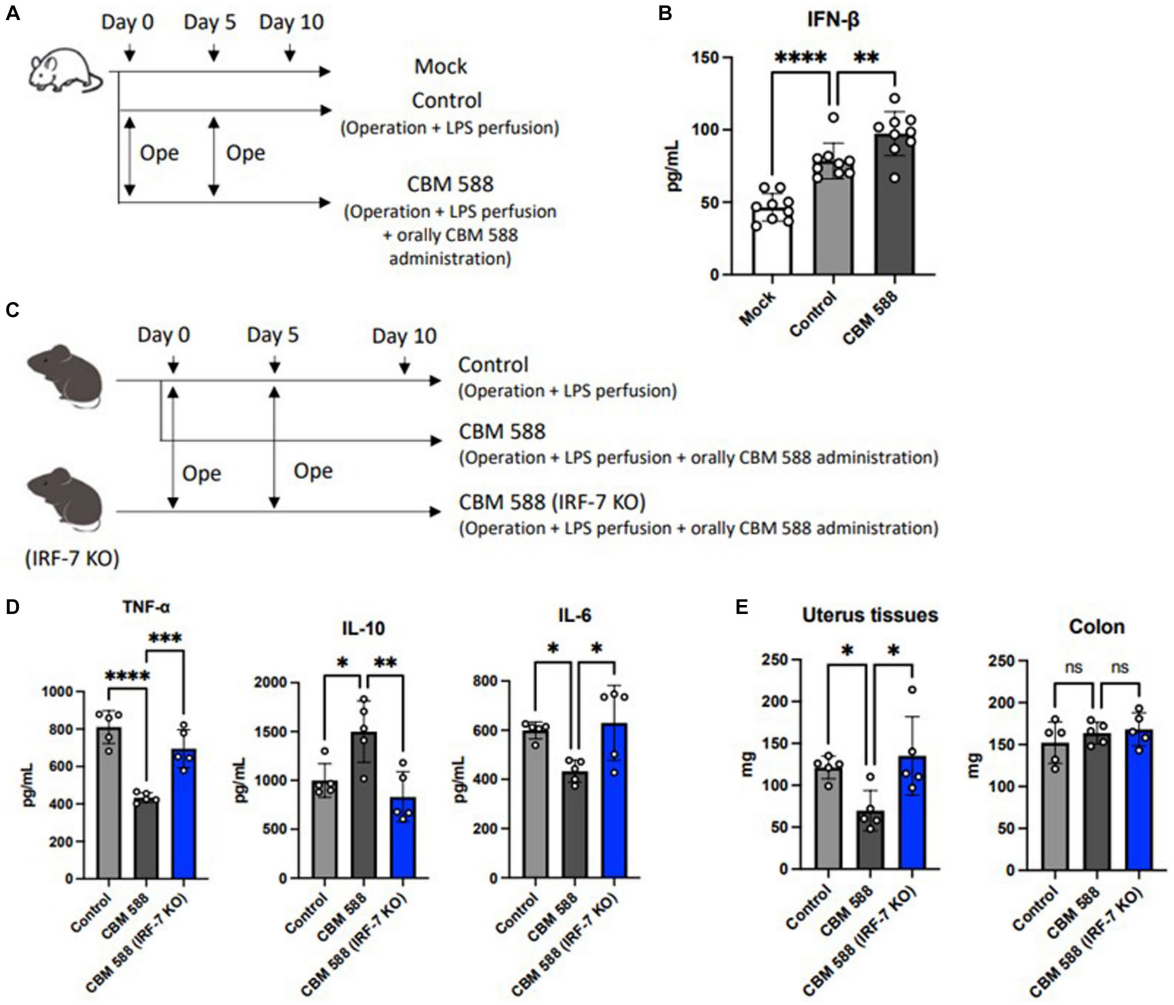
Orally administered *C. butyricum* shows anti-inflammatory effects through the interferon-β upregulation in uterine tissues. **(A)** Balb/c mice were divided 3 groups. Mock group received no treatment (*n* = 8), control group (*n* = 8) and CBM 588 group (*n* = 8) received operations on day 0 and 5. Then, control group and CBM 588 group conducted LPS perfusions from uterus horns. Only CBM 588 group received orally CBM 588 administrations from days 0 to 9. **(B)** IFN-β level in uterine tissues on day 10. **(C)** C57BL/6 J mice were divided into control group (*n* = 5), CBM 588 group (*n* = 5) and CBM 588 (IRF-7 KO) group (*n* = 5). They received operations on day 0 and 5, and then, conducted LPS perfusions from uterus horns. Two groups in the back received oral CBM 588 administrations from day 0 to 9. **(D)** Cytokine levels in uterine tissues on day 10. **(E)** Weights of uterine tissues and colon on day 10. Results are presented as mean ± standard deviation. Each dot represents a single mouse. Results were considered statistically significant when the differences were *p* < 0.05, as determined by one-way ANOVA **(B–E)**. See also Supplementary Figure S4. CBM 588, *Clostridium butyricum* MIYAIRI 588; LPS, lipopolysaccharide; IFN-β, interferon-β; IRF-7, interferon regulation factor-7.

*Lactobacillus* spp. and *Limosilactobacillus* spp. upregulated type I IFNs and enhanced bacterial clearances (Redanz and Kriegel, 2022; Sekheri et al., 2022; Wang L. et al., 2023). Hence, we detected IFN-β production in uterine tissue, and we found the upregulation of IFN-β after oral CBM 588 administrations (Figures 4A,B). Additionally, to determine the effects of type I IFNs on the anti-inflammatory effects of orally administered CBM 588 in uterine tissues, we conducted an *in vivo* study using *Irf*-7 gene knock out (KO) mice (Figure 4C). Consequently, the anti-inflammatory effects of CBM 588 were attenuated (Figure 4D). Then, the CBM 588 (IRF-7 KO) group showed higher pro-inflammatory cytokines, such as TNF-α, IL-17A, IFN-γ, and IL-6, and lower anti-inflammatory cytokines, such as IL-10, TGF-β, and IL-4, in the uterine tissues than those of the CBM 588 group (Figure 4D; Supplementary Figure S4A), thereby exaggerating uterine tissue inflammation with the inhibition of type I IFNs productions. Moreover, compared to the CBM 588 group, the weight gain of uterine tissues was significantly increased by the inhibition of type I IFNs production (Figure 4E), whereas body weights and survival rates did not show significant differences between the CBM 588 group and CBM 588 (IRF-7 KO) group (Supplementary Figures S4B,C).

### 3.5 Orally administered *Clostridium butyricum* alters lipid metabolism in uterine tissues

**Figure 5:**
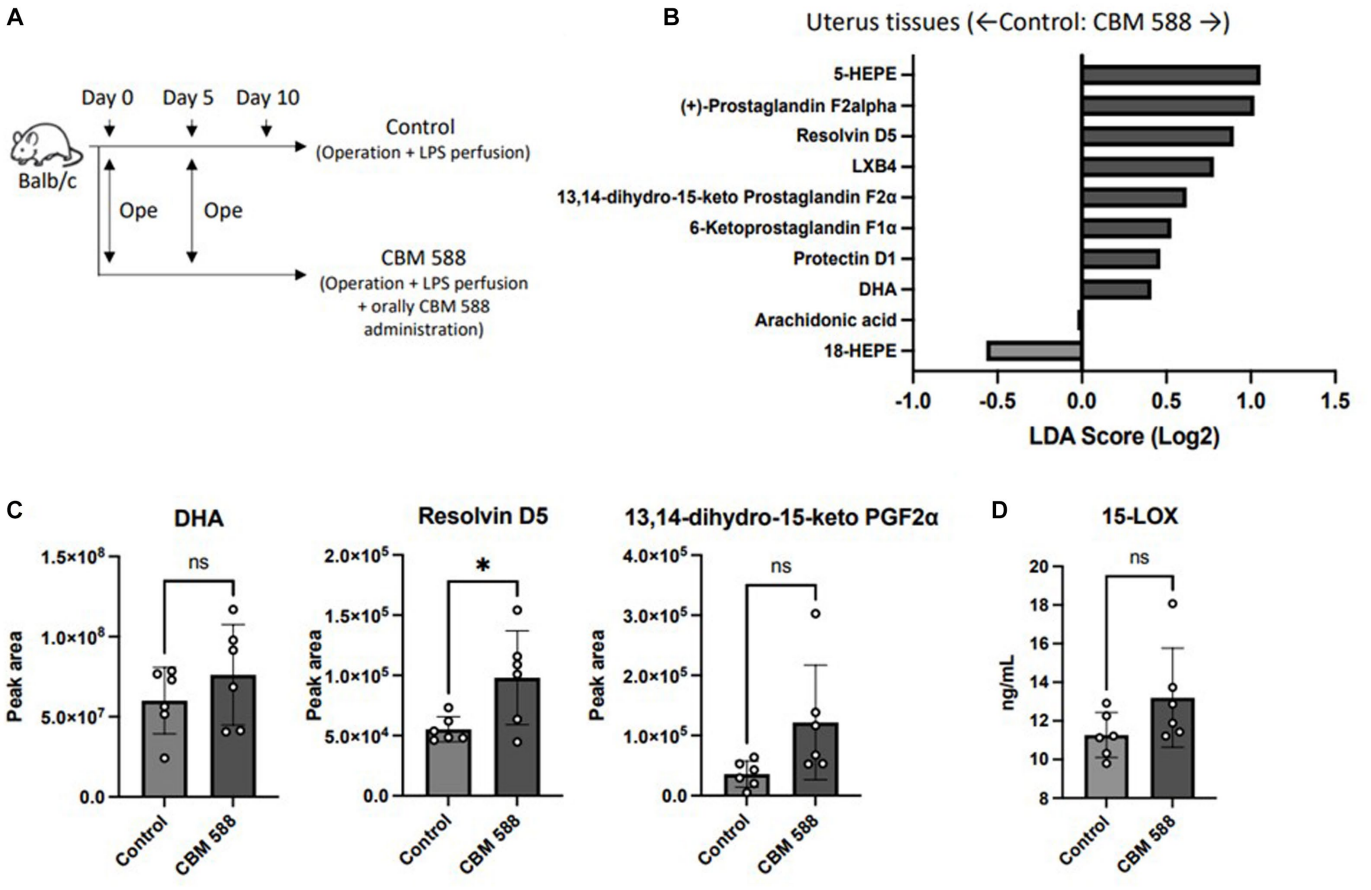
Orally administered *C. butyricum* alters lipid metabolism in uterine tissues. **(A)** Balb/c mice were divided into 2 groups. The control group (*n* = 4) and CBM 588 group (*n* = 4) received operations on days 0 and 5. The control group and CBM 588 group conducted LPS perfusions from the uterus horns. Only the CBM 588 group received oral CBM 588 administrations from days 0 to 9. **(B)** LDA Score (Log2) of lipid metabolites in uterine tissues when compared with the control and CBM 588 administration group. **(C)** Peak areas of DHA, resolvin D5 and 13,14-dyhydro-15-keto PGF2α in uterine tissues. Results are presented as mean ± standard deviation. Each dot represents an individual mouse. Results were considered statistically significant when differences were *p* < 0.05 by Student’s *t*-test **(C)**. See also Supplementary Figure S5. CBM 588, *Clostridium butyricum* MIYAIRI 588; LPS, lipopolysaccharide.

To determine how oral CBM 588 administration affects lipid metabolism in host uterine tissues, comprehensive lipid metabolite analyses were conducted (Figure 5A). Ten lipid metabolites, including structural isomers, were assigned after comparison with fragment libraries (Figure 5B). Among them, only three metabolites (docosahexonoeic acid: DHA, resolvin D5 and 13,14-dihydro-15-keto PGF2α) in the uterine tissues of the CBM 588 administration group showed less than 0.1 *p*-values compared with those of the control group (Figure 5C; Supplementary Figure S5A). Similar with a previous study (Ariyoshi et al., 2020), oral CBM 588 administration upregulated 15-lipoxygenase (15-LOX) expression (Figure 5D), and resolvin D5, which are metabolites derived from DHA (an ω-3 polyunsaturated fatty acid: PUFA) with 15-LOX (Perry et al., 2020), was significantly upregulated in the CBM 588 administration group (Figure 5C). Additionally, we conducted an *in vivo* study to reveal the impact of orally administered CBM 588-induced resolvin D5 in uterine tissues on endometritis (Supplementary Figure S5B). To inhibit the effects of ω-3 PUFAs, including resolvin D5, we used 15-LOX inhibitor (PD146176). Consequently, resolvin D5 administration showed anti-inflammatory effects, compared to control group (Supplementary Figure S5C). However, 15-LOX inhibitor attenuated the anti-inflammatory effects of resolvin D5.

### 3.6 Orally administered *Clostridium butyricum* shows anti-inflammatory effects through G protein-coupled receptor 120

**Figure 6:**
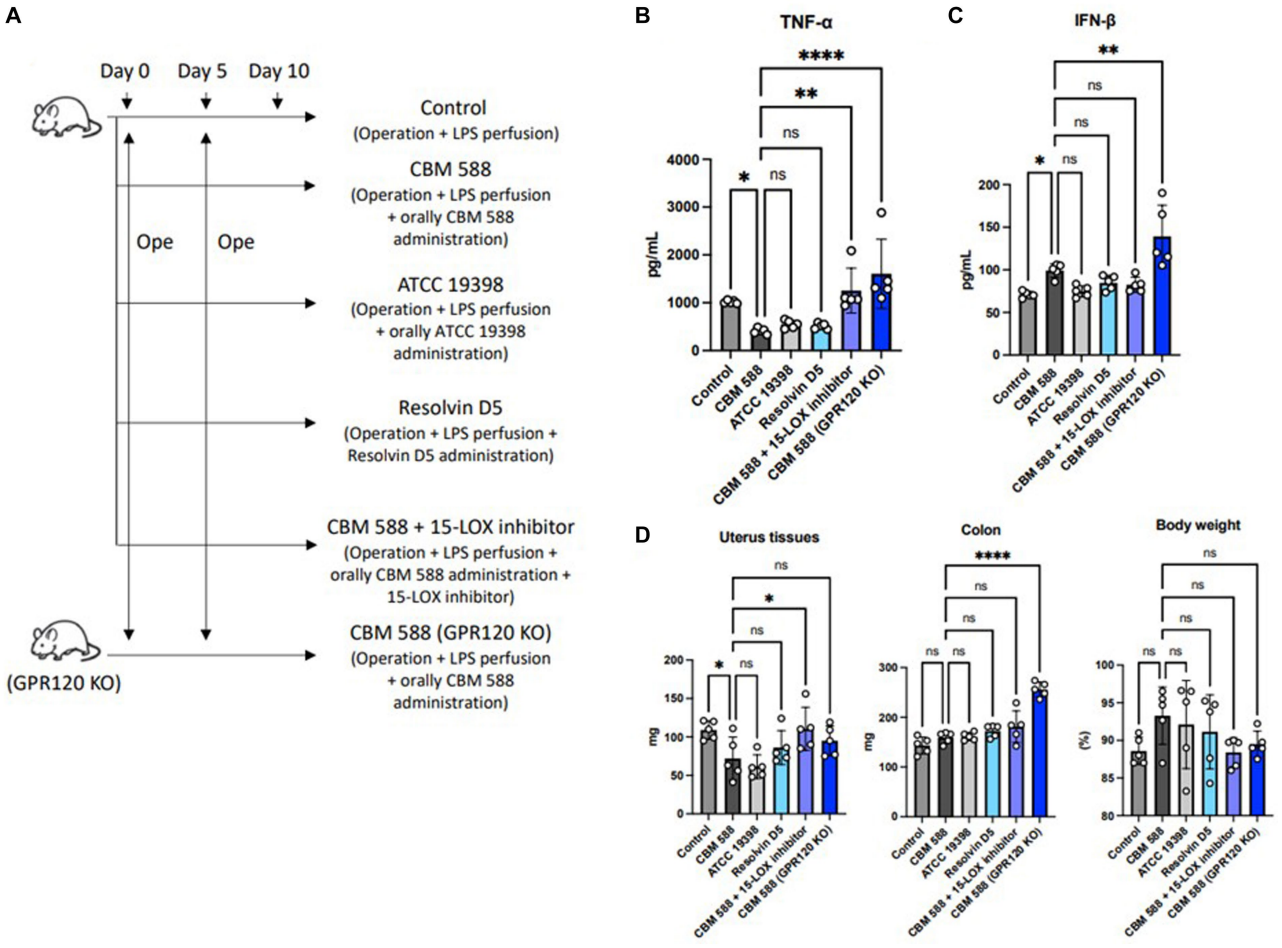
Orally administered *C. butyricum* shows anti-inflammatory effects through G protein-coupled receptor 120. **(A)** Balb/c mice were divided into 6 groups (*n* = 5, respectively). All groups received operations on days 0 and 5, and then, conducted LPS perfusions from the uterus horns. CBM 588 group, CBM 588 + 15-LOX inhibitor group and CBM 588 (GPR120 KO) group received oral CBM 588 administrations from days 0 to 9. ATCC 19398 group received oral *C. butyricum* ATCC 19398 administrations from days 0 to 9. Resolvin D5 group received resolving D5 intraabdominally from day 0 to 9. **(B)** TNF-α level in uterine tissues on day 10. **(C)** IFN-β level in uterine tissues on day 10. **(D)** Weights of uterus tissues, colon and mouse body. Results are presented as mean ± standard deviation. Each dot represents a single mouse. Results were considered statistically significant when the differences were *p* < 0.05, as determined by one-way ANOVA **(B–D)**. CBM 588, *Clostridium butyricum MIYAIRI* 588; LPS, lipopolysaccharide; IFN-β, interferon-β.

We conducted an *in vivo* study to reveal the mechanisms of orally administered CBM 588 in uterine tissues (Figure 6A). To inhibit the effects of resolvin D5, we used G-protein-coupled receptor 120 (GPR120)-expressing genes KO mice and a 15-LOX inhibitor (PD146176). In results, resolvin D5 administration showed anti-inflammatory effects similar to those of CBM 588. However, GPR 120 KO mice and the 15-LOX inhibitor attenuated the anti-inflammatory effects of orally administered CBM 588 (Figure 6B). The impacts of GPR 120 deletion on CBM 588 induced anti-inflammatory effects was greater than that of the 15-LOX inhibitor. We also found that orally administered CBM 588 showed greater anti-inflammatory effects than the *C. butyricum* reference isolate (ATCC 19398), although the differences were not significant. Additionally, the GPR 120 deletion and 15-LOX inhibitor did not decrease IFN-β productions with orally CBM 588 administrations (Figure 6C). The weight gain of uterine tissues tended to increase with GPR 120 deletion and 15-LOX inhibition (Figure 6D). Conversely, body weight showed a decreasing tendency, but there were no significant differences between the CBM 588 groups and the other groups.

## 4 Discussion

**Figure 7:**
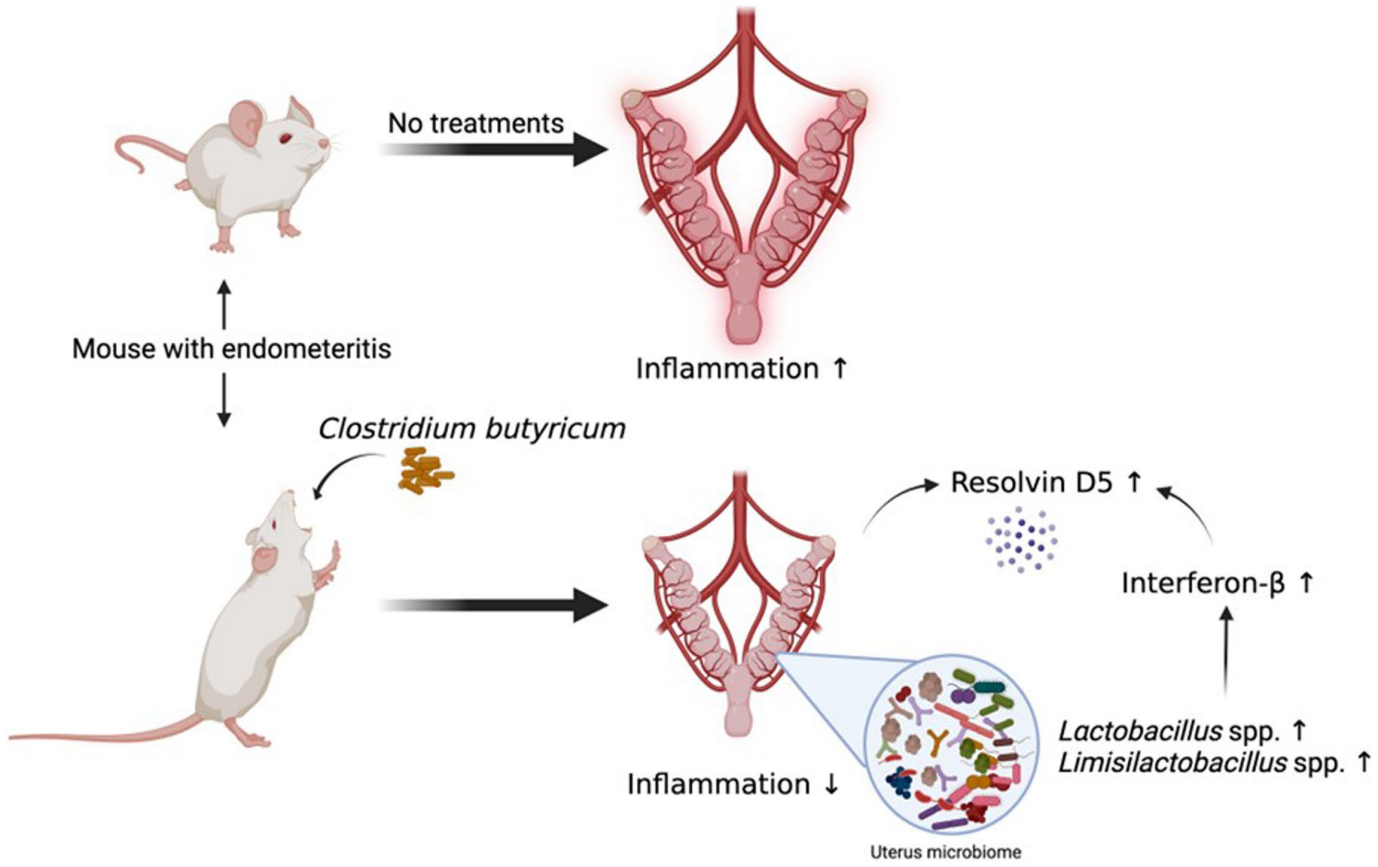
Orally administered *C. butyricum* alters uterine microbiome and lipid metabolisms to attenuate inflammation in uterus tissues. Orally administered CBM 588 can contribute to show the anti-inflammation effects in uterine tissues through the IFN-β upregulation and GPR120 activation with lipid metabolites, such as resolvin D5. CBM 588, *Clostridium butyricum* MIYAIRI 588; IFN-β, interferon-β.

In this study, we described a gut-uterine tissue axis mechanism. Orally administered CBM 588 altered uterine microbiome and induced the upregulation of some lipid metabolites, such as ω-3 PUFA resolvin D5, in uterine tissues. These effects can contribute to the anti-inflammatory effects through the IFN-β upregulation and GPR120 activation in uterine tissues (Figure 7).

*Clostridium butyricum* (ATCC 19398) showed anti-inflammatory effects in mice with endometritis when administered directly into the uterine tissues, resulting in an improvement in the birth rate of mice (Mun et al., 2022). In this *in vivo* study, we observed anti-inflammatory effects in the uterine tissues, even though CBM 588 was orally administered to mice with endometritis. These findings suggest that CBM 588 can be used to prevent and treat endometritis through noninvasive oral administration.

The commensal microbiome of the host plays an important role in maintaining homeostasis by modulating the host immune system and metabolic functions (Garrett et al., 2007; Vijay-Kumar et al., 2010; Elinav et al., 2011; Hooper et al., 2012). In our microbiome analysis of the uterine tissues, oral administration of CBM 588 altered the uterine microbiome in mice with endometritis and increased the relative abundance of *Lactbacillus* spp. *Limosilactobacillus* spp. are among the major species in the uterine microbiome of healthy volunteers (Moreno et al., 2016; Chen et al., 2017; Lozano et al., 2021).

*Lactobacillus* spp. and *Limosilactobacillus* spp. have the potential to alleviate inflammatory diseases through the inhibition of the inflammatory factors in the NF-κB and MAPKs signaling pathway (Qin et al., 2009; Peter et al., 2018; Liu et al., 2022; Zong et al., 2023). Furthermore, these species show the anti-inflammatory effects with the upregulation of type I IFNs (Bourgeois et al., 2011). Then, interferon regulation factor (IRF)-7 plays an important role in the host defense against bacterial infection by regulating IFN-β (Qing and Liu, 2023). Additionally, oral administration of CBM 588 enhanced type I and III IFNs in lung tissues through IRF-1/−7 activation, and IRF-7 affected IFNs production more effectively than IRF-1 in our previous *in vivo* study (Hagihara et al., 2022). Hence, we expected that, similar with lungs, uterine tissues were also affected with CBM 588 to produce IFN-β and attributed to show the anti-inflammatory effects. Consequently, we admitted the upregulation of IFN-β in uterine tissues with oral CBM 588 administration and attenuations of the CBM 588 induced anti-inflammatory effects in IRF-7 KO mice.

Furthermore, oral administration of CBM 588 significantly promoted resolvin D5 production in the uterine tissues of mice with endometritis, and the lipid metabolite shows anti-inflammatory effects as specialized pro-resolving mediators (SPMs) (Perry et al., 2020). Consequently, inhibitions of some lipid metabolite productions, including ω-3 PUFA resolvin D5, with 15-LOX inhibitor and diminished GPR120 expression attenuated the anti-inflammatory effects of orally administered CBM 588 in uterine tissues, whereas we did not make sure that 15-LOX inhibitor and diminishment of GPR120 would downregulate the resolvin D5 production in the CBM 588 treated mice. Therefore, we expected that orally administered CBM 588-induced resolvin D5 would play an important role in the anti-inflammatory effects in uterine tissues. Additionally, IFN-β treatments accelerates clearance of bacteria, and accelerates resolution of inflammation with concomitant increases in SPMs such as resolvin D5 (Sekheri et al., 2022). Hence, we expected that CBM 588-induced IFN-β would also contribute to promote the resolvin D5 production to show anti-inflammatory effects in uterine tissues.

However, this study has some limitations. First, we did not clarify the precise mechanisms by which CBM 588 increases *Lactobacillus* spp. and *Limosilactobacillus* spp., although we confirmed that CBM 588 is related to their proliferation. Second, to evaluate the anti-inflammatory effects of CBM 588 on uterine tissues, we used a 15-LOX inhibitor and GPR120 KO mice. However, the 15-LOX inhibitor is not specific to resolvin D5 production, which affects the production of other lipid mediators, and has anti-inflammatory effects (Perry et al., 2020). Additionally, GPR120 is the main ligand for ω-3 unsaturated fatty acid receptor and it is not specific to only resolvin D5 (Chiang et al., 2012; Husted et al., 2017). Hence, our results suggest the possibilities that other lipid mediators may play important roles in these effects. Related to the limitation, we did not confirm that the absence of GRP120 or IRF-7 genes inhibits the resolvin D5-induced anti-inflammatory effects on the uterine tissue or not. Hence, further study to clear the relationships between GRP120 or IRF7 genes and resolvin D5-induced anti-inflammatory effects are needed. Thirdly, CBM588 has anti-inflammatory effects in the lung since the increase of IFN-λs through the upregulation of lipid metabolites and GPR120 activation (Hagihara et al., 2022). However, compared to the type I IFNs, IFN-λs (type III) have not cleared the roles for the responses to the inflammation in uterine tissues. Hence, we focused on the role of type I IFNs in this study. Additionally, among the GRP120 KO mouse, IFN-β production was not inhibited but increased in mice treated with CBM588, compared to the control group. We expected that this result derives from the facts that there are some mechanisms to stimulate IFNs productions (Lazear et al., 2015). However, we could not reveal the precise mechanism in this study. Hence, further study is needed to clear the precise mechanism and roles of CBM 588-induced IFN-λ in uterine tissues with inflammations. Finally, the human microbiome differs from the mice microbiome (Human Microbiome Project Consortium, 2012). Further studies are required to investigate whether these results can be replicated in humans.

## 5 Conclusion

In summary, orally administered CBM 588 showed anti-inflammatory effects in the uterine tissues. Orally administered CBM 588 affected the uterine microbiome, and the relative abundances of *Lactobacillus* spp. and *Limosilactobacillus* spp. were upregulated. These changes can lead to the upregulation of IFN-β and altered host lipid metabolic functions in uterine tissues. Notably, the anti-inflammatory lipid metabolite resolvin D5 in uterine tissues was upregulated by oral CBM 588 administrations. However, the anti-inflammatory effects derive from oral CBM 588 administration was attenuated with the deletion of GPR 120 and 15-LOX inhibition. Our results reveal a gut-uterine tissue axis mechanism and provide novel insights into targets for endometritis treatment and prophylaxis.

## Data availability statement

The datasets presented in this study can be found in online repositories. The names of the repository/repositories and accession number(s) can be found in the article/Supplementary material.

## Ethics statement

The animal studies were approved by the Ethics Committee of Aichi Medical University. The studies were conducted in accordance with the local legislation and institutional requirements. Written informed consent was obtained from the owners for the participation of their animals in this study.

## Author contributions

MH: Conceptualization, Data curation, Formal analysis, Investigation, Methodology, Project administration, Validation, Visualization, Writing – original draft, Writing – review & editing. TA: Investigation, Writing – review & editing. SE: Investigation, Writing – review & editing. KO: Funding acquisition, Writing – review & editing. MT: Funding acquisition, Writing – review & editing. HK: Investigation, Writing – review & editing. YS: Investigation, Writing – review & editing. TU: Investigation, Writing – review & editing. TM: Investigation, Writing – review & editing. NaM: Investigation, Writing – review & editing. JH: Investigation, Writing – review & editing. NA: Investigation, Writing – review & editing. NoM: Investigation, Writing – review & editing. HM: Funding acquisition, Resources, Writing – review & editing.

## Footnotes

^1^https://arriveguidelines.org

^2^https://www.avma.org

^3^https://www.jalam.jp/

^4^Selleckchem.com
