# Supplementary material for: Oral Clostridium butyricum on mice endometritis through uterine microbiome and metabolic alternations
Source: Front Microbiol. 2024 Feb 21;15:1351899. doi: 10.3389/fmicb.2024.1351899 (PMC10915095; doi:10.3389/fmicb.2024.1351899)
Supplement: Supplementary file 1 [file Data_Sheet_1.pdf]

Supplemental data

Title: Oral *Clostridium butyricum* on mice endometritis through uterine microbiome and metabolic alternations

Mao Hagihara, Tadashi Ariyoshi, Shuhei Eguchi, Kentaro Oka, Motomichi Takahashi, Hideo Kato, Yuichi Shibata, Takumi Umemura, Takeshi Mori, Narimi Miyazaki, Jun Hirai, Nobuhiro Asai, Nobuaki Mori, Hiroshige Mikamo

Figure S1

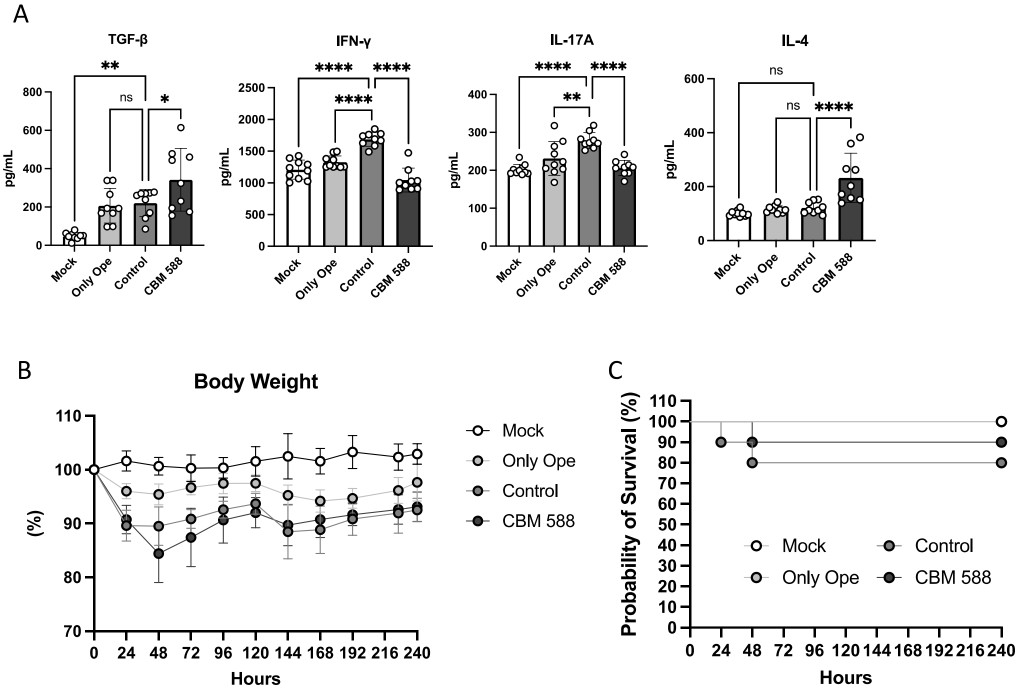

Figure S1. Orally administered *C. butyricum* shows anti-inflammatory effects in uterine tissue

(A) Balb/c mice were divided 4 groups. Mock group received no treatment, only ope group, control group and CBM 588 group received operations on day 0 and 5. Control group and CBM 588 group conducted LPS perfusions from uterus horns at same timings. Cytokines in uterus tissues on day 10 (n = 9-10).

(B and C) Body weights (B) and survival ratio (C).

Results are presented as mean ± standard deviation (A and B). Each dot represents an individual mouse (A). Results were considered statistically significant when differences were $p < 0.05$ (****$p < 0.0001$, ***$p < 0.001$, **$p < 0.01$, *$p < 0.05$; ns indicates not significant) by one-way ANOVA (A), two-way ANOVA (B), or the Kaplan-Meier method (C).

CBM 588, *Clostridium butyricum* MIYAIRI 588

LPS, lipopolysaccharide

Figure S2

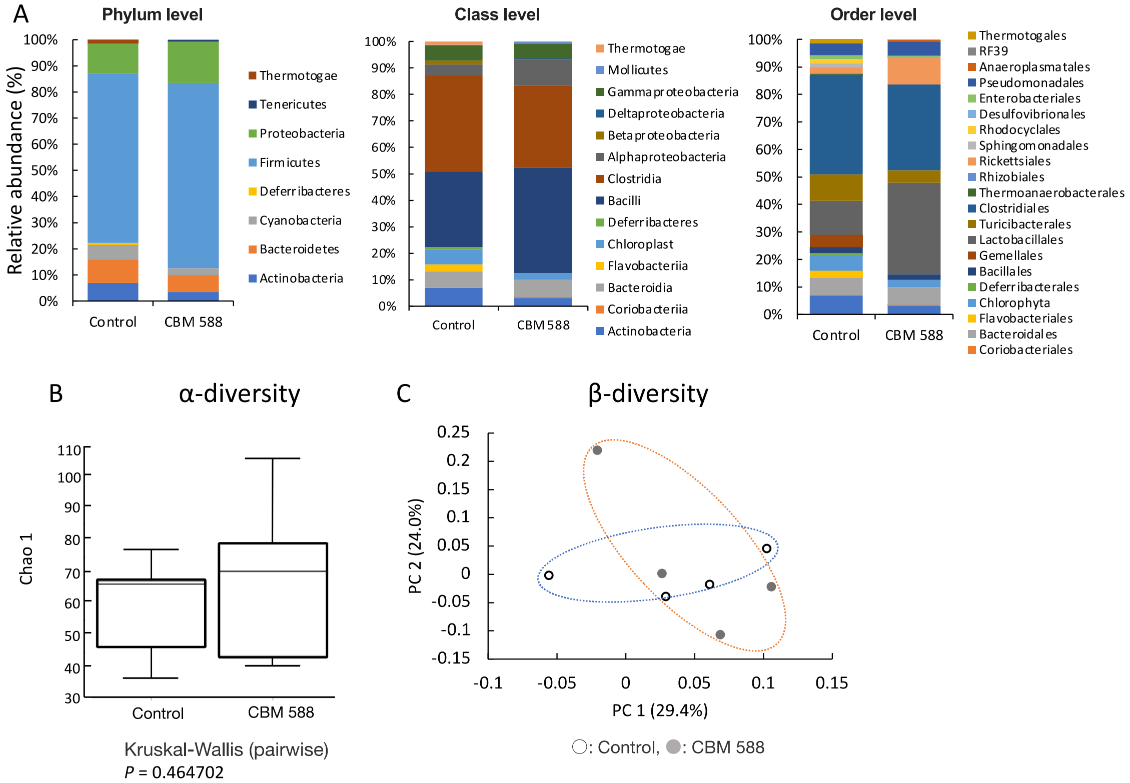

Figure S2. Orally administered *C. butyricum* alters uterine microbiome

(A) Balb/c mice were divided 2 groups. Control group (n = 4) and CBM 588 group (n = 4) received operations on day 0 and 5. Control group and CBM 588 group conducted LPS perfusions from uterus horns at the same timings. Bacterial composition in uterus tissues at the phylum level, class level, order level. Data are represented with mean value of relative abundances (%) for each group.

(B) Comparison of the Chao 1 index of different groups (n = 4, respectively). The box and whiskers represent the smallest and largest values, with the median in the center of each box.

(C) PCoA based on weighted Unifrac distances between control group and CBM 588 group. Each dot represents an individual mouse.

CBM 588, *Clostridium butyricum* MIYAIRI 588

LPS, lipopolysaccharide

Figure S3

A

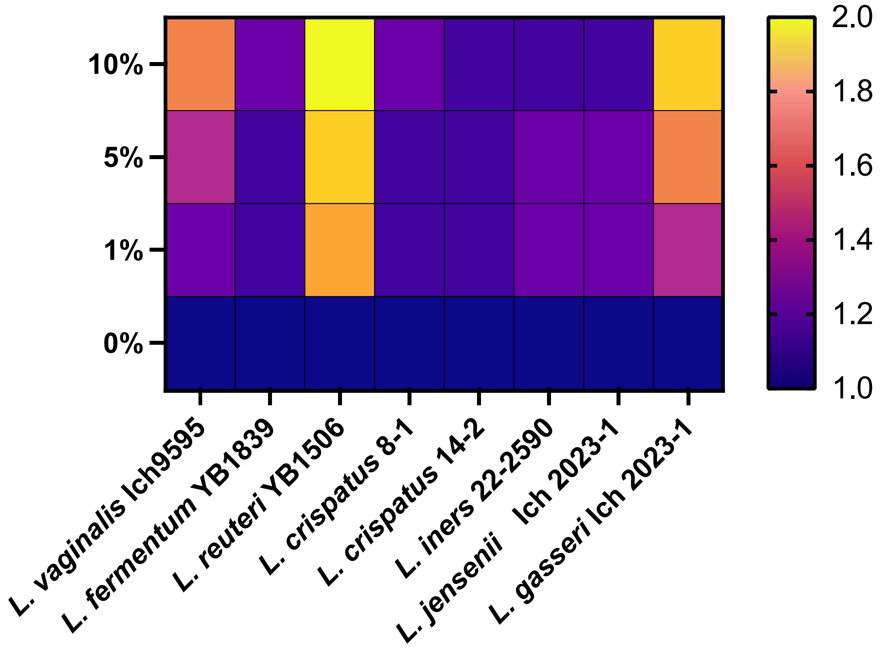

Figure S3. The supernatant of *C. butyricum* enhances *Lactobacillus* spp.*, and Limosilactobacillus* spp. proliferations with *in vitro* study

(A) *Lactobacillus* spp. and *Limosilactobacillus* spp. were exposed to the supernatant of *C. butyricum* (CBM 588) incubation medium (0, 1, 5, and 10%) and incubate anaerobically. Heatmap represents the ratio of the bacterial concentrations after 24 h incubation (1%, 5%, and 10% CBM 588 supernatants were added to each bacterial suspension / bacterial suspension without CBM 588 supernatant [0%]).

CBM 588, *Clostridium butyricum* MIYAIRI 588

Figure S4

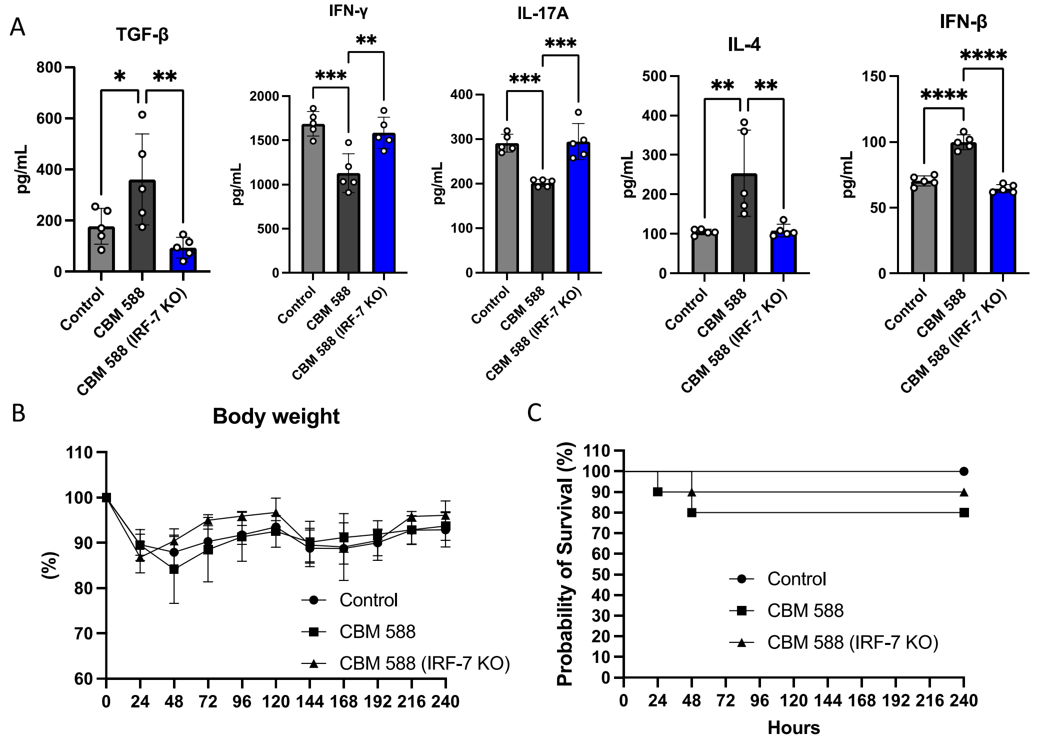

Figure S4. Orally administered *C. butyricum* induced IFN-β shows anti-inflammatory effects

(A) C57BL/6J mice were divided into control group (n = 5), CBM 588 group (n = 5) and CBM 588 (IRF-7 KO) group (n = 5). They received operations on day 0 and 5, and then, conducted LPS perfusions from uterus horns. Two groups in the back received orally CBM 588 administrations from day 0 to 9.

(B and C) Body weights (B) and survival ratio (C).

Results are presented as mean ± standard deviation (A and B). Each dot represents an individual mouse (A). Results were considered statistically significant when differences were $p < 0.05$ (****$p < 0.0001$, ***$p < 0.001$, **$p < 0.01$, *$p < 0.05$) by one-way ANOVA (A), two-way ANOVA (B), or the Kaplan-Meier method (C).

CBM 588, *Clostridium butyricum* MIYAIRI 588

LPS, lipopolysaccharide

Figure S5

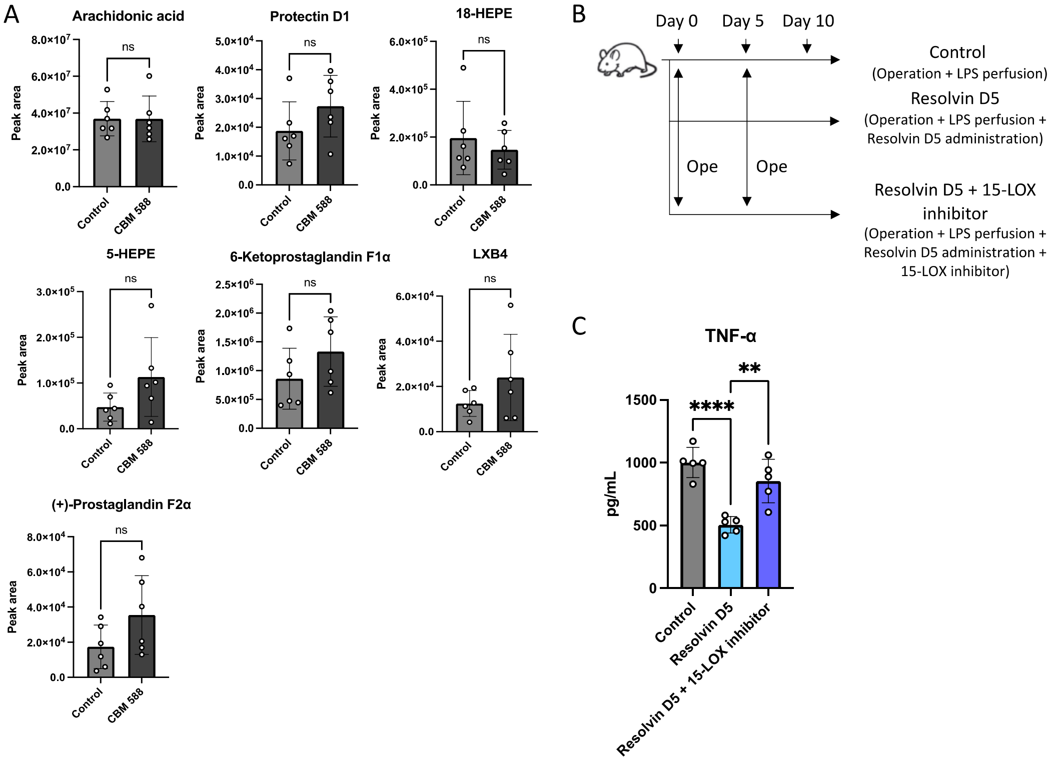

Figure S5. Orally administered *C. butyricum* alters lipid metabolism in uterine tissues

(A) Balb/c mice were divided 2 groups. Control group (n = 6) and CBM 588 group (n = 6) received operations on day 0 and 5. Control group and CBM 588 group conducted LPS perfusions from uterus horns at the same timings. Only CBM 588 group was received orally CBM 588) administrations from day 0 to 9.

(B) Balb/c mice were divided into 3 groups (n = 5, respectively). All groups received operations on days 0 and 5, and conducted LPS perfusions from the uterus horns. Resolvin D5 group and resolvin D5 + 15-LOX inhibitor group received resolving D5 intraabdominally from day 0 to 9.

(C) TNF-α level in uterine tissues on day 10.

Results are presented as mean ± standard deviation (A and C). Each dot represents an individual mouse (A and C). Results were considered statistically significant when differences were $p < 0.05$ (****$p < 0.0001$, **$p < 0.01$; ns indicates not significant) by Student's *t*- test (A) and one-way ANOVA (C).

CBM 588, *Clostridium butyricum* MIYAIRI 588

LPS, lipopolysaccharide

Data availability statements

The microbiome data have been deposited in the DDJB (PRJDB16701) and are publicly available as of the date of publication. The LC-MS/MS data have been deposited in the DDBJ (MTBKS234) and are publicly available as of the date of publication.
